# Strain-Specific Phosphate Mobilization in Enterobacter: Organic Acid Production and Genomic Architecture of Solubilization Mechanisms

**DOI:** 10.3390/ijms27010322

**Published:** 2025-12-27

**Authors:** Ekaterina Alexeevna Sokolova, Inna Viktorovna Khlistun, Olga Viktorovna Mishukova, Irina Nikolaevna Tromenschleger, Evgeniya Vladimirovna Chumanova, Elena Nikolaevna Voronina

**Affiliations:** 1 Institute of Chemical Biology and Fundamental Medicine, Siberian Branch of the Russian Academy of Sciences, Novosibirsk 630090, Russia; 2 Department of Molecular Biology and Biotechnology, Faculty of Natural Sciences, Novosibirsk State University, Novosibirsk 630090, Russia

**Keywords:** phosphate-solubilizing microorganisms, mechanism of phosphate solubilization, syntenic analysis of the genomes, malic acid

## Abstract

Phosphate-solubilizing microorganisms (PSMs) show promise for sustainable agriculture, yet inconsistent field performance limits their application. We investigated phosphate solubilization mechanisms in *Enterobacter ludwigii* strains GMG278, GMG291, GMG378 and *Enterobacter soli* GMG1156 through greenhouse wheat experiments, high-performance liquid chromatography (HPLC) organic acid analysis, and comparative genomics. Greenhouse trials demonstrated that bacterial inoculation compensated for phosphorus deficiency, with GMG291, GMG1156, and GMG278 showing superior performance. HPLC identified malic acid as the predominant secreted organic acid, with *E. soli* producing threefold higher concentrations than *E. ludwigii* strains. Phosphate solubilization efficiency followed the order FePO_4_ > AlPO_4_ > Ca_3_(PO_4_)_2_, with maximal release (95.9–97.7 μg/mL) from iron phosphate despite lower malic acid secretion, suggesting siderophore involvement. An inverse correlation between malic acid levels and soluble phosphate concentrations likely reflects competitive bacterial phosphate uptake and secondary precipitation processes. Comparative genomics revealed missense mutations in the LuxR transcriptional regulator of strain GMG378 (Asp86Asn and Arg97Leu) near predicted DNA-binding domains, correlating with reduced solubilization capacity. Phosphate solubilization in Enterobacter proceeds primarily through metal–malic acid complex formation, with strain-specific efficiency linked to LuxR-regulated biofilm formation genes. These findings suggest PSM screening should incorporate biofilm-related genetic markers alongside acid production measurements.

## 1. Introduction

Phosphorus is the second most essential nutrient for plants, playing a crucial role in their growth and development. Only phosphorus dissolved in soil as the phosphate anion is directly available to plants [1]. However, even in fertile soils, the concentration of soluble phosphorus does not usually exceed 10 μM, due to its high reactivity with calcium (Ca), iron (Fe) and aluminum (Al), which leads to precipitation of the phosphate anion in the form of salts. Soluble phosphorus deficiency is a common problem, and phosphorus fertilizers are widely applied to maintain crop yields. Nevertheless, even when phosphorus fertilizers are used, approximately 75–90% of the applied phosphorus is precipitated as FePO_4_, AlPO_4_ and Ca_3_(PO_4_)_2_ [2]. Consequently, new applications of fertilizers are made each year to maintain adequate phosphorus levels, and the overall concentration of phosphorus in many soils has significantly increased over time [3]. According to theoretical estimates, the phosphorus already accumulated in agricultural soils is enough to maintain maximum crop yields worldwide for approximately 100 years [1]. Therefore, if we could find a way to utilize the reserves of “fixed phosphorus”, we could meet the needs of plants both from what has already accumulated and by increasing the efficiency of newly introduced phosphorus through fertilizers.

Phosphate-solubilizing microorganisms are present in soils and have the potential to play an important role in supplying plants with phosphorus. Although many microorganisms exhibit the ability to dissolve phosphate in laboratory settings, the outcomes of field trials vary significantly. This inconsistency hinders the widespread use of PSMs in agriculture. Various explanations for this inconsistency have been suggested, but a comprehensive understanding of the underlying biochemical and molecular processes involved in phosphorus dissolution is essential to identify the root cause. These processes likely differ among different PSM types, resulting in different effects on bacteria under field conditions.

Proposed mechanisms for the solubilization of inorganic phosphorus include soil acidification processes, chelation, and exchange reactions. The pH reduction hypothesis was formulated and supported by the results of an experiment with Rhizobium, which showed that phosphate dissolution is a consequence of the formation of 2-ketogluconic acid. The hypothesis of acidification of the medium was supported by the fact that the phosphorus-dissolving activity of Rhizobium was eliminated by the addition of NaOH [4]. However, there are some questions about how this mechanism works under conditions of high soil buffer capacity. It seems unlikely that organic acids secreted by bacteria in relatively small amounts could significantly shift the soil pH without other factors. Additionally, the ability of other microorganisms and fungi to lower pH does not always correlate with their ability to dissolve mineral phosphates [2].

The second proposed mechanism involves the ability of organic acids, such as acetate, lactate, malate, oxalate, succinate, citrate, gluconate, and ketogluconate, to form complexes with calcium, iron, and aluminum ions [5]. Chelating properties are important for the process of phosphate solubilization. The importance of these chelating properties is confirmed by an experiment in which adding 0.05 M EDTA to a medium resulted in a similar solubilizing effect as inoculating with Penicillium bilaii [6]. Gluconic acid is one of the best-studied solubilization agents and is formed through the PQQ-dependent glucose dehydrogenase system. This system includes the genes responsible for PQQ biosynthesis (pqqA-pqqG) and structural glucose dehydrogenase genes (gdhA and gdhB) [7]. The organic acids of the tricarboxylic acid cycle, such as citrate, malate, and succinate, are also formed through specific enzyme systems that are regulated by ATP/ADP ratios and substrate availability. Switching between oxidative and reductive modes of TCA (tricarboxylic acid cycle) operation, as well as the activation of anaplerotic CO2 fixation pathways through pyruvate carboxylase and phosphoenolpyruvate carboxylase, play a critical role in the accumulation of organic acids [8].

Alternative mechanisms of phosphate solubilization have been described, including the production of inorganic acids, exopolysaccharides, and siderophores [2]. It is likely that different microorganisms employ different mechanisms, either individually or in combination. For example, *Bacillus megaterium* releases phosphorus from organic phosphates, but does not dissolve mineral phosphates [6,9]. Different forms of inorganic phosphorus are dissolved to varying degrees by PSM, depending on the type of microorganism. Experimental results from *Bacillus subtilis*, *Bacillus* sp., *Penicillium* sp., and *Aspergillus* sp. show that Ca_3_(PO_4_)_2_ is dissolved very effectively, while AlPO_4_ is dissolved to a lesser extent. Some strains of *Bacillus* sp., *Chaetomium nigricolor*, and *Aspergillus* sp. are unable to transfer soluble phosphorus from AlPO_4_ into solution in significant amounts [10].

Among the bacterial genera that have the ability to solubilize phosphates, Enterobacter stands out for its diverse metabolic strategies. This genus belongs to the Enterobacteriaceae family and has facultative anaerobic metabolism, allowing it to adapt to different soil conditions. Unlike obligate aerobes like Pseudomonas and anaerobes like Lactobacillus, members of the genus Enterobacter can switch between respiratory and fermentative pathways depending on oxygen availability. Recent studies have found that different Enterobacter species can produce multiple organic acids at once. For instance, E. hormaechei produces nine different acids, including gluconic, glutamic, ketoglutaric, malic, and succinic [11]. The Enterobacter sp. ITCB-09 strain exhibits high phosphate-solubilizing capacity through the production of organic acids [12]. However, the molecular mechanisms underlying the differences in solubility between different strains of the same species are not yet fully understood.

Previously, in our laboratory, screening tests provided data on the different abilities of phosphate solubilization among various strains of *Enterobacter ludwigii*, including GMG278 (53.97 ± 16.58 μg/mL), GMG291 (106.82 ± 22.64 μg/mL) and GMG378 (53.66 ± 13.45 μg/mL) [13], as well as on the high-level phosphate solubilization capacity of *Enterobacter soli* strain GMG1156 (134.69 ± 14.8 μg/ mL) [14]. These differences between closely related strains present a unique opportunity to identify the genetic factors that determine the effectiveness of phosphate solubilization. To test this hypothesis and understand the mechanisms of phosphate solubilization used by *Enterobacter* sp., we investigated the impact of these strains on wheat growth under conditions of phosphorus deficiency and performed HPLC analysis to measure the secretion of tricarboxylic acid organic acids during culture in the presence of different types of insoluble phosphate (AlPO_4_, FePO_4_, Ca_3_(PO_4_)_2_). Additionally, comparative genomic analysis was conducted to identify genetic variations that correlate with phenotypic differences between strains.

## 2. Results

### 2.1. The Effect of Inoculation with Enterobacter ludwigii GMG278, GMG291, and GMG378 and Enterobacter soli GMG1156 Strains on the Growth and Development of Wheat Plants in a Greenhouse Assay

**Figure 1:**
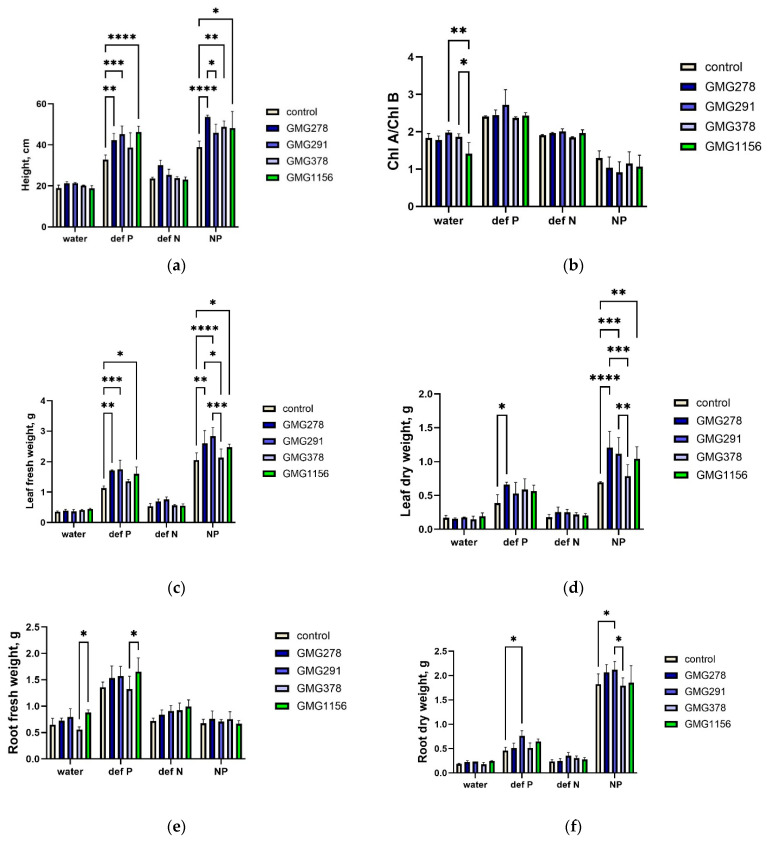
Characteristics of wheat in an experiment of cultivation in conditions of phosphorus and nitrogen deficiency. (**a**) Plant height (cm), (**b**) Chl A/Chl B, (**c**) Leaf fresh weight (g), (**d**) Leaf dry weight (g), (**e**) Root fresh weight (g), (**f**) Root dry weight (g). * *p* < 0.05, ** *p* < 0.01, *** *p* < 0.001, **** *p* < 0.0001.

The differences in the practical impact of phosphate solubilization, which were previously identified in laboratory tests for *Enterobacter ludwigii* GMG278, GMG291, and GMG378 and *Enterobacter soli* strain GMG1156 [13,14], were assessed as part of an experiment in a greenhouse setting on wheat plants. Figure 1 and Table A1 present the results of measurements on wheat plants grown under these conditions.

Based on the data obtained, we can see that inoculation with a bacterial suspension under phosphorus-deficient conditions helped plants achieve growth similar to that of plants receiving full nutrition (from 38.8 ± 7.1 cm to 46.2 ± 2.8 cm vs. control 32.8 ± 2.2 cm under P-deficient conditions; from 45.9 ± 4.0 cm to 53.7 ± 0.8 cm vs. control 38.9 ± 2.9 cm in full nutrition condition). While the height of wheat under water-only conditions varied from 18.8 ± 1.3 cm to 21.3 ± 0.3 cm during inoculation, and it was 18.9 ± 1.4 cm without inoculation.

The weight of fresh leaves in conditions of phosphorus deficiency during inoculation varied from 1.35 ± 0.07 g to 1.74 ± 0.30 g and was statistically significantly higher than in the absence of inoculation (1.13 ± 0.08 g). These indicators were closer to those in conditions of full nutrition (from 2.13 ± 0.28 g to 2.2 ± 0.28 g with inoculation vs. 2.05 ± 0.24 g in control). Under conditions of water-only irrigation, the indicators were much lower (from 0.36 ± 0.07 g to 0.44 ± 0.03 g with inoculation) and independent of inoculation (0.35 ± 0.02 g in control).

The best compensation for phosphorus deficiency was observed in plants in experiments with *Enterobacter ludwigii* GMG278, GMG291, and *Enterobacter soli* GMG1156. In contrast, growth rates under nitrogen-deficient conditions were similar to those under conditions of complete nutrient deficiency (Table A1).

**Figure 2:**
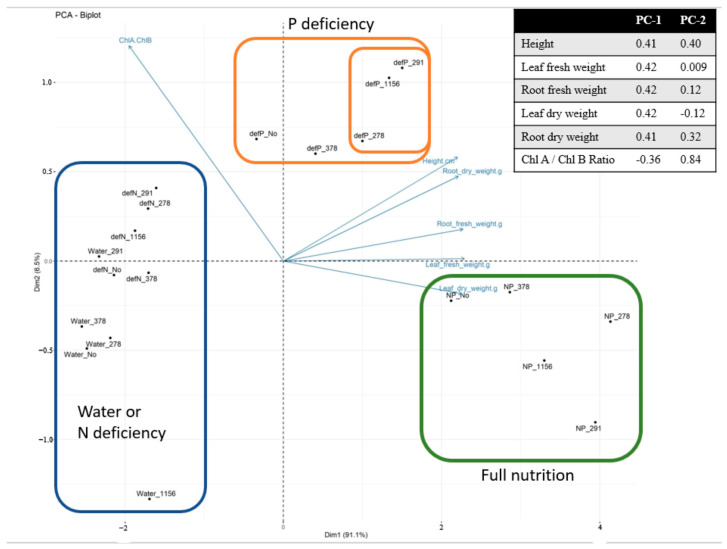
Biplot based on the analysis of the principal components of growth and biochemical characteristics of wheat grown under nutrient-deficient conditions.

For visualization, a PCA was performed and a biplot was built (Figure 2).

The samples were clustered into three clusters: the first group had complete nutrient deficiency or nitrogen deficiency only (blue contour), the second group had phosphorus deficiency (orange contour), and the third group had complete nutrition (green contour). All nitrogen-deficient samples were grouped together with samples that had minimal nutrient conditions, indicating that there was no compensation for the nitrogen deficiency. Since the principal component 1 (PC1) accounts for 91.1% of the variance in the parameter analysis, the main separation between samples is along the *x*-axis. It can be seen that samples GMG291, GMG1156, and GMG278 (the inner orange contour) are located on the right side of the axis, indicating that they are closer to the group with full nutrition than inoculated GMG378 or those without inoculation. Under full-nutrition conditions (green contours), these same samples are positioned further to the right along the *x*-axis, consistent with their enhanced growth promotion effect even under optimal nutrition conditions compared to non-inoculated controls.

### 2.2. Effect of Malic Acid Secretion by Studied Strains on AlPO_4_, Ca_3_(PO_4_)_2_, and FePO_4_ Solubilization

**Figure 3:**
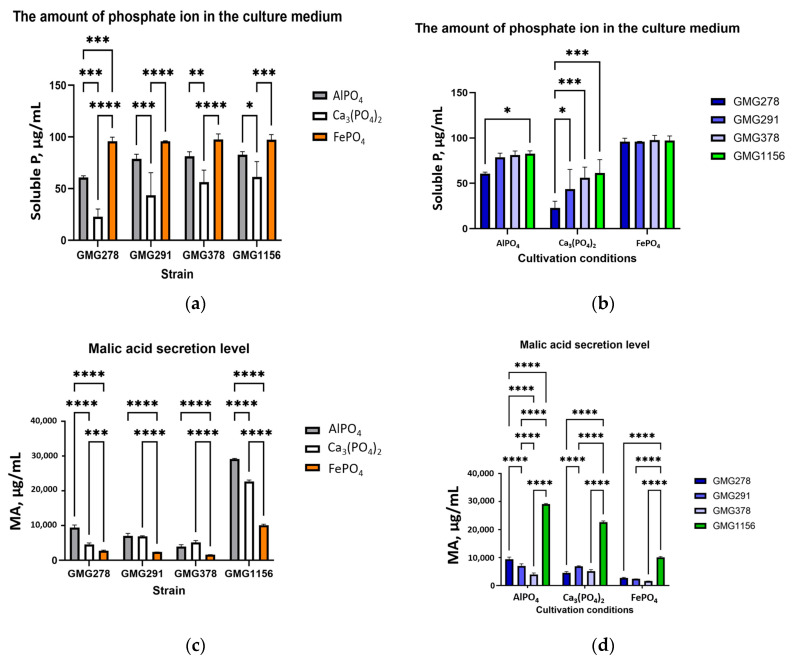
Levels of secretion of malic acid and soluble phosphate in an in vitro experiment: (**a**) The amount of phosphate ion in the culture medium by strain; (**b**) The amount of phosphate ion in the culture medium by salt; (**c**) MA secretion by strain; (**d**) MA secretion by salt. * *p* < 0.05, ** *p* < 0.01, *** *p* < 0.001, **** *p* < 0.0001.

**Figure 4:**
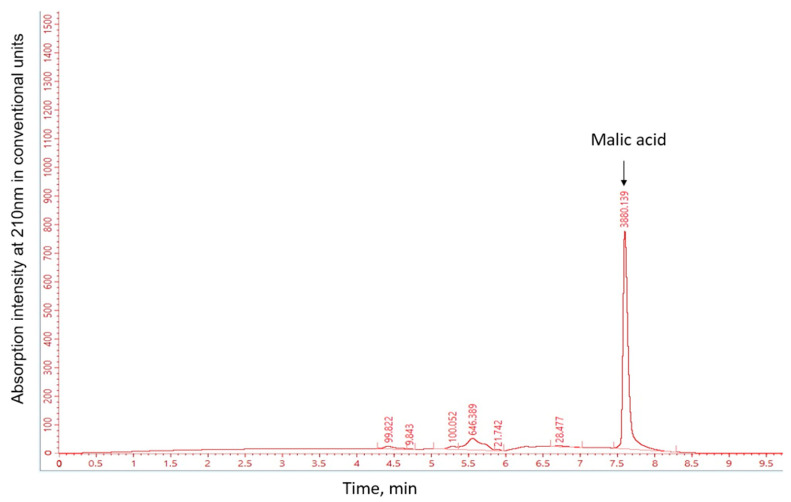
An example of a chromatogram showing the separation of tricarboxylic acids using HPLC. The main peak in the graph corresponds to the malic acid peak.

Since three insoluble salts, AlPO_4_, Ca_3_(PO_4_)_2_ and FePO_4_ act as sources of inorganic phosphate in nature, the ability to solubilize phosphate and the potential mechanism of this process were investigated by growing the studied strains on a minimal medium supplemented with one of these salts. The pH of the culture medium, the concentration of phosphate ions, and the level of TCA cycle organic acids were experimentally evaluated (Figure 3 and Table A2). According to the HPLC results for the detection of organic acids, malic acid was the only major component (Figure 4), so further analysis focused on it.

According to the results obtained, the most significant acidification of the medium occurred in the presence of FePO_4_, with a pH ranging from 2.65 to 2.75. The pH of the medium decreased to 3.4 to 4.2 in the presence of calcium and aluminum phosphates. The highest levels of phosphate ions in the culture fluid were found for samples cultured with FePO_4_, while slightly lower values were observed for AlPO_4_ and Ca_3_(PO_4_)_2_ (Figure 3a,b).

**Figure 5:**
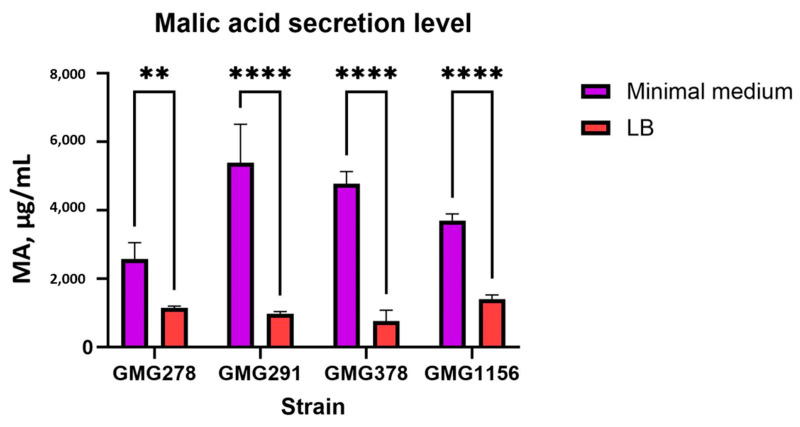
The level of secretion of malic acid under cultivation conditions in a minimal medium and LB. ** *p* < 0.01, **** *p* < 0.0001.

*Enterobacter soli* strain GMG1156 secretes malic acid in significantly higher amounts compared to all *Enterobacter ludwigii* strains under similar conditions. *E. soli GMG1156* secreted approximately threefold more malic acid (Figure 3c,d). In the presence of FePO_4_, the malic acid secretion levels for all strains are lower than in the presence of AlPO_4_ and Ca_3_(PO_4_)_2_ (Figure 3c,d). A clear trend is observed for *Enterobacter ludwigii*, with the level of malic acid secretion being approximately 30–40% lower for strain GMG378 compared to GMG278 and GMG291. To verify the non-random nature of this trend, we measured the malic acid secretion level during cultivation of the strains on minimal and rich LB media. According to the results (Figure 5 and Table A3), the secretion levels increased by 2–5 times for all strains in the minimal environment. However, there is variability in the malic acid secretion among *Enterobacter ludwigii* strains. GMG378 showed the minimum secretion level, while GMG291 exhibited the maximum. Tricalcium phosphate was used as a source of phosphorus in a minimal medium. The relative differences in malic acid secretion among *E. ludwigii* strains remained consistent with the previous experiment (see Table A2).

The phosphate ion level in experiments with AlPO_4_ and Ca_3_(PO_4_)_2_ was inversely correlated with the level of malic acid secretion, contrary to expectations based on the chelation mechanism (where higher organic acid levels should produce more soluble phosphate). The minimum phosphate ion level among *Enterobacter ludwigii* strains was recorded for GMG278, while the maximum level was for GMG378. In the presence of FePO_4_, there was no difference in the phosphate ion levels between different strains.

### 2.3. Search for Genetic Determinants in the Complete Genomes of the Studied Strains That May Explain Their Differences in Phosphate Solubilizing Ability

#### 2.3.1. Search for Major Genomic Differences Between *E. ludwigii* and a Reference Strain, as Well as Among *E. ludwigii* Strains Themselves

**Figure 6:**
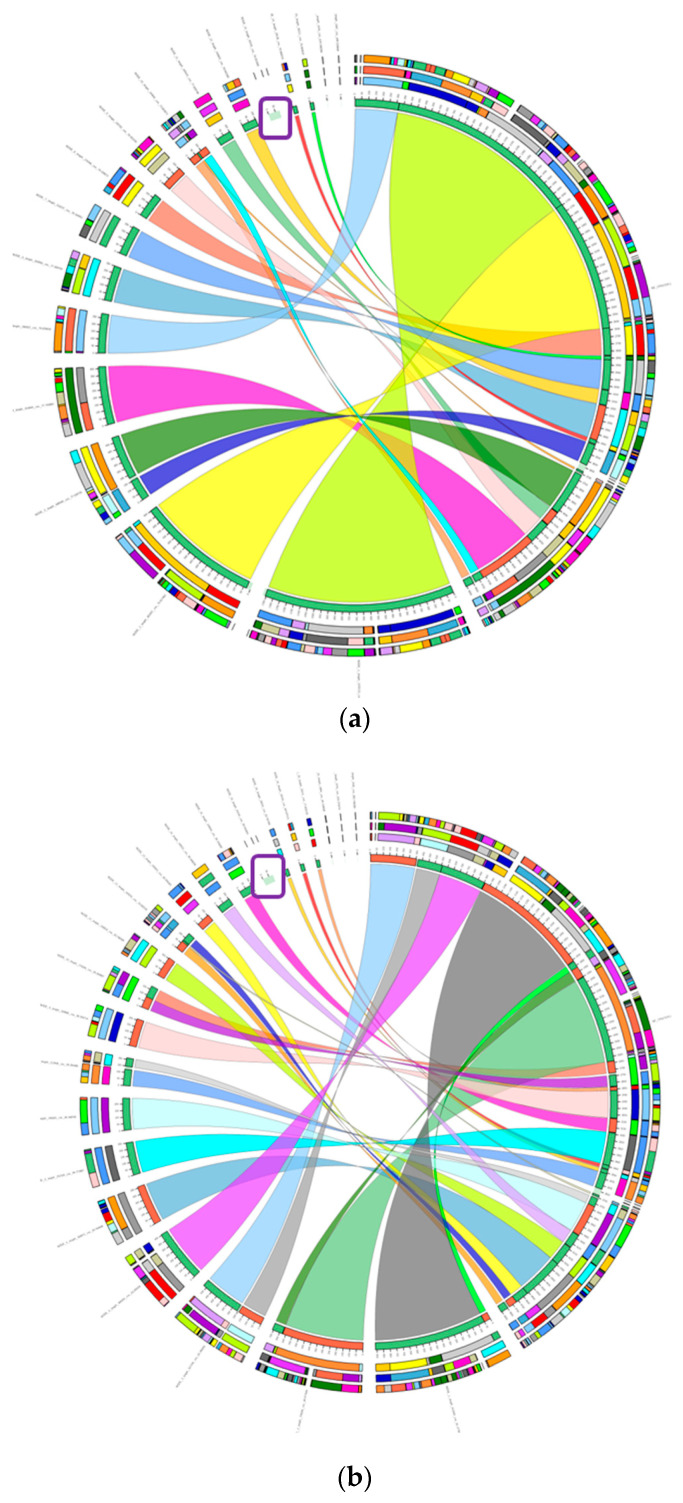

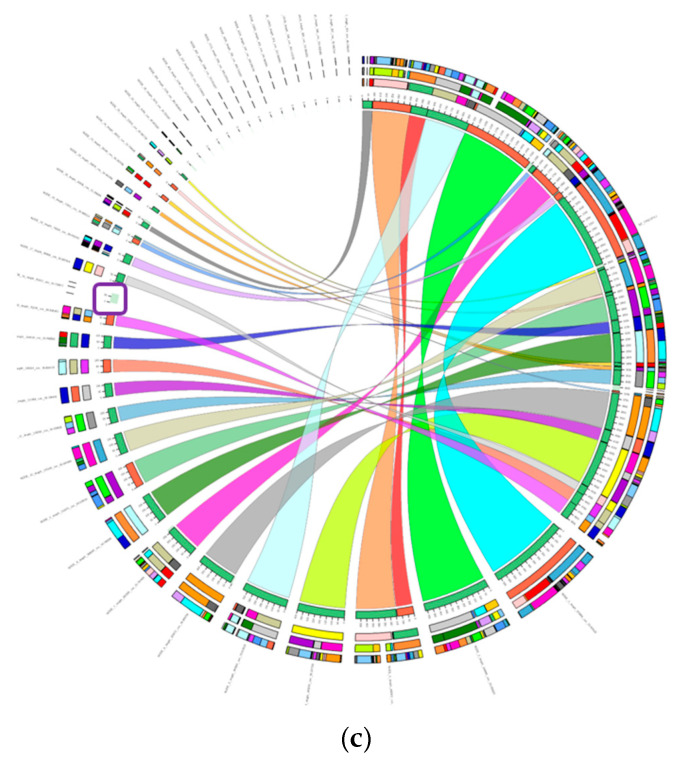
Circos plots of the synteny analysis of large rearrangements in the studied strains compared to the reference *Enterobacter ludwigii* strain EN-119. (**a**) Comparison with *Enterobacter ludwigii* GMG278 as the reference; (**b**) Comparison with *Enterobacter ludwigii* GMG291 as the reference; (**c**) Comparison with *Enterobacter ludwigii* GMG378 as the reference. These graphs visualize the differences in a region of the genome from two strains that are superimposed on top of each other. All aligned areas are connected with colored lines. Unaligned areas remain separate. In this image, a 81 kilobase (kb) long section is highlighted with a purple box, which was not present in the reference strain’s chromosome. The same colors indicate the zones that are aligned by the Sibelia program between the strains.

The search for large genomic rearrangements was performed using the Sibelia program, followed by Circos visualization (Figure 6). Based on the results of the comparison, a region of the genome from the studied strains was identified that was not present in the chromosomal genome of the reference strain. The length of this section was 81,012 base pairs (bp) for GMG291 and GMG378, and 81,016 bp for GMG278.

This region has been annotated by the PROKKA program. It was found to be completely identical in all three strains and includes the list of genes listed in Supplemental Table S1. To determine which microorganisms contain this genomic region in the same sequence, BLAST (version v2.12.0) alignment was performed using the prok_nt database. The BLAST results showed that this region is present on the chromosome of several *Enterobacter* sp., including *E. ludwigii*, *E. cloacae*, *E. bugandensis*, *E. chuandensis*, *E. asburiae*, *E. roggenkampii*, and *E. kobei*. However, for the *Enterobacter ludwigii* strain EN-119, this sequence aligns with the sequence of plasmid pEN-119.

Thus, the studied strains did not differ from each other in terms of large genomic rearrangements. Therefore, the difference in phosphate solubilization between the strains could not be explained by the acquisition or loss of a significant portion of the genome.

#### 2.3.2. Search for SNPs, Indels in *E. ludwigii* Genomes GMG278, GMG291 vs. GMG378

**Figure 7:**
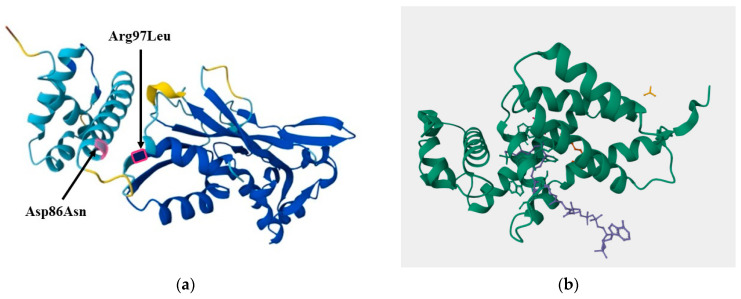
The structure of the *LuxR* protein. (**a**) The predicted structure from AlphaFold v2.3.2 is shown, with substitutions marked that change the amino acid class of amino acids Asp86Asn and Arg97Leu. Color coding represents prediction confidence (pLDDT score): dark blue (>90, very high confidence), light blue (70–90, confident), yellow (50–70, low confidence), orange (<50, very low confidence). (**b**) The structure predicted from AlphaPill is shown, indicating the locations of DNA strands and TF activator molecules.

Based on the greenhouse experiment for wheat cultivation, strains GMG278 and GMG291 were identified as having a high ability to solubilize phosphate, while strain GMG378 was identified as having a reduced ability. This greenhouse experiment was used as the basis for the analysis, rather than the results from plate assays, as greenhouse experiments are more representative of real field conditions than plate assays. The analysis to identify the causes of the differences was based on the assumption that GMG278 and GMG291 strains should have the same genetic context regulating phosphate solubilization processes, while the GMG378 strain has the opposite. The search was performed using the Snippy program, followed by a comparison of the resulting VCF files. Initially, all variants that contained any possible differences between GMG378 and either GMG278 or GMG291 were filtered out, leaving a total of 47,068 positions. Then, the substitutions were sorted into two separate lists based on their type: missense_variant and intergenic_variant. All synonymous substitutions were excluded, presumably because they were considered unimportant. In the second stage of the filtration process, all genes with unknown functions were removed. After filtering, 43,950 variants remained, of which 57 were missense mutations in genes of known function (see Supplemental Table S2). The list of genes that have experienced a missense mutation includes 22 different genes. Among these 22 genes, we selected all the genes previously described as being involved in phosphate metabolism, as well as all transcription factors. After that, we manually analyzed the list of transcription factors and conducted a search to determine whether they are involved in the regulation of the phosphate metabolism genes. Among these, there is one gene directly related to phosphate metabolism, *PhoE*, as well as two transcription factors, *TetR* and *LuxR*. In the *PhoE* and *TetR* proteins, amino acid substitutions occur within the same class of amino acids, so it is likely that the protein domains are not disrupted. However, in *LuxR*, two amino acid substitutions involve changes between different amino acid classes, from Asp86 to Asn and Arg97 to Leu (Figure 7).

Among the intragenic variants, a search was conducted for genes potentially involved in malic acid production and secretion. These include *mdh* (malate dehydrogenase) [15], *fumA*, *fumB*, *fumC* (fumarase), *arcA*, and *FNR* [16], isocitrate lyase and malate synthase [17]. Two intergenic SNPs were identified in the vicinity of the transcriptional regulator FNR: n.1270193C>T and n.1270427A>C.

#### 2.3.3. Search for SNPs, Indels in the Genomes of *E. ludwigii* GMG278, GMG291, GMG378 vs. *E. soli* GMG1156

The search was performed using the Snippy program, followed by filtering out only those positions that were the same for all *E. ludwigii* strains (GMG278, GMG291, and GMG378) and different for the *E. soli* strain (GMG1156). A total of 325,102 positions were identified. Next, the positions located in or near genes with a known function were selected (299,139 positions). Only missense substitutions were left in the final list (384 positions, see Supplementary Table S3). We did not analyze intergenic variant type substitutions when comparing strains of different species. In the list of missense substitutions, genes that may be functionally involved in phosphate solubilization processes were selected (Supplementary Table S4). The *PhoE* phosphoporin gene contains 20 missense substitutions, 10 of which change the amino acid class. The LysR transcription factor has 4 amino acid (AA) mutations, three of which change the AA class. FrsA (fermentation/respiration switch protein) has 14 missense mutations, 8 of which change the class. Despite the large number of AK class changes, the predicted 3D structure of these proteins does not appear to change.

## 3. Discussion

### 3.1. Experimental Validation of the Efficacy of Phosphate Solubilization in a Controlled Environment

The results of the greenhouse experiment on wheat have led to three key conclusions regarding the ability of the Enterobacter strains studied to solubilize phosphate in conditions similar to those found in soil. Firstly, inoculation with the *E. ludwigii* strains GMG278, GMG291, and GMG378 did not compensate for nitrogen deficiency, consistent with their lack of a cluster of nitrogenase genes and confirming the specificity of their physiological activity. Secondly, all strains exhibited the ability to compensate for a deficiency in soluble phosphate forms, as indicated by plants achieving growth rates comparable to those of fully fertilized controls. Thirdly, strains of the *E. ludwigii* species demonstrated varying efficiencies, with GMG278 and GMG291 providing significantly better phosphate compensation compared to GMG378 despite their high genetic similarity.

The PCA has clearly visualized this separation, with the samples forming three distinct clusters: complete nutrient deficiency, phosphorus-deficient with bacterial inoculation, and complete nutrition. Strains GMG278, GMG291, and GMG1156 are located closer to the complete nutrition cluster, along principal component 1 (PC1), which explains 91.1% of the variation. It is important to note that our chosen experimental model, with a limited supply of phosphorus in the peat substrate and a predominance of insoluble phosphate forms, is as close as possible to real-world soil conditions. This is crucial, as one of the major challenges in the research on phosphate-solubilizing microorganisms is the low reproducibility of laboratory findings in field trials [6].

It is important to note that the experiments were conducted under controlled greenhouse conditions using a peat-based substrate. Typically, the number of introduced microbes decreases rapidly after they are introduced into the soil. The survival rate of the inoculated strain depends on several factors, including the composition, temperature, humidity, pH level, availability of substrate, and competition from other microorganisms [18]. This last factor is particularly significant, as the decrease observed in non-sterile soil can often be eliminated in sterile soil [19]. In order to select strains that are suitable for field use, it is essential to conduct experiments under non-sterile, field conditions as well.

A comparison of the results from phosphate solubilization screening and greenhouse experiments revealed several significant discrepancies. Based on the 96-well plate screening, the strain GMG291 exhibited almost twofold greater phosphate solubilization capacity (106.82 μg/mL) than GMG278 and GMG378 (53.97 and 53.66 μg/mL, respectively). *E. soli* strain GMG1156 demonstrated the highest values (134.69 μg/mL), while the GMG278 strain showed relatively low values in the 96-well plate screening but compensated for phosphorus deficiency in the greenhouse at a level similar to GMG291 and GMG1156. These discrepancies between quantitative indicators of in vitro phosphate solubilization and practical effects on plants suggest that the absolute level of soluble phosphate produced under standardized laboratory conditions may not fully explain strain effectiveness in complex soil systems. This observation highlighted the importance of understanding the molecular and biochemical processes that underlie the differences between the strains under study, which necessitated a detailed examination of the mechanisms of phosphate solubilization implemented by Enterobacter species.

The mechanisms underlying PGPR-plant interactions involve multiple interconnected processes beyond direct phosphate supply, including alteration of rhizosphere chemistry, production of plant growth regulators, competitive exclusion of pathogens, and modulation of plant stress responses. Our findings emphasize that effective phosphate solubilization requires not only biochemical capacity (organic acid production) but also spatial organization capacity—the ability to form biofilms that concentrate solubilizing agents at the mineral-root interfaces. Understanding PGPR-plant interactions thus requires moving beyond reductionist assessments of single traits toward evaluating how multiple traits are spatially and temporally integrated in structured soil environments.

### 3.2. The Mechanism of Phosphate Solubilization In Vitro: The Role of Organic Acids and Siderophores

#### 3.2.1. Malic Acid as the Primary Solubilizing Agent

Based on the results of high-performance liquid chromatography for the detection of organic acids, malic acid was the only major organic acid detected in the culture medium of all strains examined; therefore, further analysis focused on malic acid. This contrasts with the characteristic profile observed in most phosphate-solubilizing bacteria, which typically exhibit a complex mixture of multiple acids. For instance, the concurrent production of multiple organic acids has been previously reported for the genus *Enterobacter*, with *E. hormaechei* PSB6 producing nine different acids (gluconic, glutamic, ketoglutaric, pyruvic, oxalic, malic, succinic, acetic, and fumaric) [11], and *Enterobacter* sp. 15S produces five primary acids (citrate, fumarate, α-ketoglutarate, malate, and oxalate) [20].

The exclusive detection of malic acid in the strains under study may be attributed to the sampling time, as various organic acids tend to accumulate at different stages of bacterial growth [21]. It is also plausible that the culture conditions facilitated the utilization of the reductive branch of the tricarboxylic acid (TCA) cycle, leading to malate accumulation under phosphate stress.

The level of malic acid secretion was significantly dependent on the cultivation conditions. All strains showed an increase in the amount of malic acid secreted in minimal medium, ranging from 2- to 5-fold higher compared to a rich LB medium (see Table A3 and Figure 5). This suggests that the production of organic acids is stimulated in response to nutrient limitation, which is consistent with activation of the Pho-regulon in conditions of phosphate deprivation [22]. Specific patterns of malic acid secretion were observed depending on the insoluble phosphate compound present. With FePO_4_, malic acid secretion by all strains was lower than with AlPO_4_ and Ca_3_(PO_4_)_2_ (see Figure 3a). Among *Enterobacter ludwigii* strains, there was a clear trend showing that strain GMG378 secreted approximately 30–40% less malic acid compared to strains GMG278 and GMG291.

#### 3.2.2. Mechanisms of Phosphate Release

To understand the mode of action of malic acid, reference data was analyzed that characterizes the complexes formed between malic acid and aluminum, iron, and calcium (see Table 1).

Based on the tabular data, Fe(III) has the highest complexation constant (15.21 ± 0.25), while Ca(II) has the lowest complexation constant. This is consistent with the results for soluble phosphorus measured for FePO_4_ and Ca_3_(PO_4_)_2_, as shown in Table 2. The highest levels of phosphate ion in the culture fluid were observed for samples cultured with FePO_4_ (95.9–97.7 μg/mL), followed by AlPO_4_ (60.8–82.8 μg/mL). The lowest levels were found for Ca_3_(PO_4_)_2_ (23.0–61.5 μg/mL), as shown in Table A2, Figure 3d. Higher complexation constants correlate with greater phosphate release.

However, for AlPO_4_ and Ca_3_(PO_4_)_2_, the complexation constants do not vary significantly (logK_1_ = 4.52 and 2.25, respectively), while the release of phosphate varies significantly. This can be attributed to differences in the solubility product constant of the initial phosphates. The solubility product constant for AlPO_4_ (Ksp = 5.75 × 10^−19^) is ten orders of magnitude greater than that of Ca_3_(PO_4_)_2_ (Ksp = 2 × 10^−29^). This facilitates the dissolution of aluminum-containing minerals even with less efficient chelation.

With regard to the role of acidification, there was a correlation between the observed pH values and the effectiveness of solubilization. The most significant acidification of the medium occurred in the presence of FePO_4_, with pH values ranging from 2.65 to 2.75. In the presence of Ca_3_(PO_4_)_2_ and AlPO_4_, the pH of the medium decreased to between 3.4 and 4.2. The acid dissociation constants (pKa) for malic acid are 3.46 and 5.71, which generally corresponded with the observed pH values. However, despite similar organic acid profiles, pH values differed significantly among the three phosphate salts. This suggests that the primary mechanism involves the formation of complexes between metal cations and malic acid, with the pH being a consequence of the number of protons released during metal-malate complex formation. If the primary mechanism were simply acidification, then the pH would be expected to be the same across all three systems, determined solely by the concentration of organic acids.

Therefore, phosphate solubilization occurs primarily through metal-malate complex formation, which displaces phosphate ions from the mineral structure and releases protons, thereby decreasing pH.

#### 3.2.3. The Role of Siderophores in Iron Phosphate Solubilization

The consistent level of soluble phosphate observed among all four strains in the FePO_4_ experiment (95.9–97.7 μg/mL, Table A2) is noteworthy, as it differs from the variations observed for AlPO_4_ and Ca_3_(PO_4_)_2_. Furthermore, the level of malic acid secretion was minimized specifically in the presence of FePO_4_ (Figure 3a), suggesting the involvement of a supplementary mechanism for extracting phosphate from insoluble FePO_4_ salt. This mechanism may involve the sequestration of iron ions by siderophores, with the release of phosphate ions occurring as a secondary process.

Genome analysis revealed that all the strains studied possess genes for the synthesis of enterobactin (Table 2), including genes for biosynthetic (*entA*, *entB*, *entC*, *entD*, *entE*, and *entF*) and transport (*entE*) pathways, as well as genes involved in the import of the iron-enterobactin complex (*fepA*, *fepB*, *fepC*, *fepD*, *fepE*, *fepF*, and *fepG*). This finding is consistent with previous reports that enterobactin is the predominant siderophore among members of the Enterobacteriaceae family, with a Fe^3+^-binding affinity in the order of 10^25^ M^−1^, one of the highest values among known biological ligands [27].

It is worth noting that the *E. soli* strain GMG1156 has an additional cluster of genes involved in the transport of iron dicitrate (*fecB*, *fecC*, *fecD*, and *fecE*) that is absent in the *E. ludwigii* strains (see Table 2). This Fec system is responsible for the transport of Fe^3+^ dicitrate complexes and can operate independently of enterobactin, providing an additional pathway for iron acquisition [28]. The presence of this additional transport system in *E. soli* may partly explain its greater efficiency in phosphate solubilization, although all strains tested achieved comparable levels of phosphate release in the presence of FePO_4_.

Among the three insoluble phosphate forms, iron phosphate released the greatest number of phosphate ions.

#### 3.2.4. Inverse Correlation of Malic Acid and Phosphate Ion Levels

One of the most surprising findings of this study was the inverse relationship between the levels of malic acid and phosphate ions in the culture media for experiments with AlPO_4_ and Ca_3_(PO_4_)_2_. The lowest level of soluble phosphorus among the *Enterobacter ludwigii* strains was observed for GMG278 (60.8 μg/mL for AlPO_4_, 23 μg/mL for CaPO_4_), while the highest level was recorded for GMG378 (81.3 μg/mL for AlPO_4_, 56.4 μg/mL for CaPO_4_) (see Table A2). Simultaneously, the highest malic acid secretion level was noted for GMG278 (9415.8 μg/mL for ALPO_4_) and the lowest for GMG378 (3980.3 μg/mL). This finding contradicts a straightforward model in which stronger chelation should result in greater phosphate release. We propose several non-exclusive explanations for this observation:

(A) The processes of reprecipitation and adsorption of released phosphorus. Despite the initial solubilization of insoluble phosphorus compounds, the formation of secondary phosphate minerals (strengite, variscite) or phosphorus adsorption on secondary iron(III) hydroxide (Fe(OH)_3_) and aluminum(III) hydroxide (Al(OH)_3_) surfaces formed during the complexation of metals with organic acids may reduce measured phosphate concentrations. Measurements were carried out at a single time point (264 h), and it is possible that strains with higher levels of malic acid may reach peak concentrations of soluble phosphorus earlier, followed by a phase of precipitation or biological uptake.
(B) Biological utilization of phosphate and uptake of organic acids. Under conditions of phosphate limitation, genes encoding proteins involved in phosphate transport (*pstSCAB-phoU*) and outer membrane pore proteins (*phoE*) are induced by the Pho regulon, as well as phosphomonoesterases and phosphodiesterases [29]. This activation of phosphate uptake systems can lead to competition for phosphate between bacteria and analytical assays. Additionally, when carbon sources are limited, bacteria may reabsorb some secreted organic acids to meet energy needs, reducing their extracellular concentration. The production of malic acid in Enterobacter occurs through two primary metabolic pathways that are optimized for different growth conditions: the tricarboxylic acid cycle (TCA) and the glyoxylate shunt. In the event of phosphate starvation, metabolic processes are disrupted as follows: phosphate deficiency results in a decrease in ATP synthesis by H^+^-ATPase, which in turn activates ArcA/B regulatory proteins and, consequently, results in the repression of TCA cycle genes and increased secretion of organic acids [30].
(C) Differences in Spatial Organization of the Process. The efficiency of solubilization may depend not only on the amount of secreted organic acids but also on the ability to create high concentrations at the mineral surface. If different strains have different capacities to form dense cellular aggregates or biofilms on the surface of insoluble phosphates, this could lead to differences in the effective concentration of organic acids at the reaction site, regardless of the overall concentration in the medium.

In summary, the in vitro analysis of solubilization mechanisms suggests that malic acid is the primary agent in this process, acting through its formation of complexes with metal cations (aluminum, iron, calcium). This leads to the release of phosphate ions from the initial compound. Additionally, siderophores may play a significant role in the solubilization of iron phosphate by specifically chelating Fe^3+^ ions.

However, the inverse relationship between malic acid levels and soluble phosphate demonstrates that phosphate solubilization effectiveness depends not only on the absolute amount of organic acids secreted but also on other factors, such as the spatial organization of the solubilization process. Bacteria that can create localized high concentrations of chelating agents at mineral surfaces may be more effective. This hypothesis is supported by recent research demonstrating the importance of bacterial biofilm formation on phosphate rock surfaces for efficient phosphate solubilization [31], as discussed in the following section.

### 3.3. Genomic Analysis Confirms the Important Role of Biofilms

The genomic analysis of *E. ludwigii* strains with contrasting phosphate solubilization phenotypes aimed to identify the molecular basis for the observed differences. Despite the absence of significant structural rearrangements between the GMG278/GMG291 (highly efficient) and GMG378 (less efficient) strains, the focus was on point mutations in functionally important genes. Out of the 57 missense mutations identified in 22 genes, the changes in the *LuxR* transcription factor were of particular interest. Two amino acid substitutions (Asp86Asn and Arg97Leu), which involved changes between amino acid classes, were localized near functionally crucial domains for binding to DNA and regulatory molecules, according to structural modeling. The discovery of mutations in the *LuxR* gene highlighted the potential role of biofilms in phosphate solubilization, which was a crucial finding for our research.

LuxR family proteins were first identified by Engebrecht in 1983 during the analysis of quorum-sensing genes. These proteins regulate bacterial survival, reproduction, virulence, and biofilm formation. The target genes regulated by LuxR can be divided into three categories based on their affinity for the protein. Class I genes have the lowest binding affinity for LuxR, requiring the highest concentration of this protein for regulation. Class II genes have an intermediate affinity, requiring a medium level of LuxR. Class III genes have the highest affinity, allowing for regulation with the lowest concentration of LuxR [22].

In 2019, Ghosh, Barman, and Mandal published research on Burkholderia that also emphasized the significance of biofilm formation. The authors made three significant observations in their work that align with our findings, leading to a general theory. First, they used scanning electron microscopy to demonstrate the development of biofilms on various phosphate rocks. Second, biofilm formation was significantly reduced with an increase in soluble phosphate in the cultivation medium. At a concentration of 500 μg/mL of K_2_HPO_4_, biofilm formation was almost completely inhibited. Lastly, structural variations in biofilm morphology were observed depending on the K_2_HPO_4_ concentration. Thick biofilm structures with maximal substrate coverage were observed in the presence of 25 μg/mL K_2_HPO_4_; as the salt concentration increased, the thickness and coverage of the substrate by the biofilm decreased [31].

These data may explain the central paradox in our results—the inverse correlation between levels of malic acid and soluble phosphate. The formation of biofilms seems to be a significant factor, given the high buffering capacity of soils. Without compartmentalization, effective medium acidification for phosphate solubilization cannot occur. In contrast, the ability to form biofilms on insoluble phosphate forms allows for the creation of local conditions that increase the concentration of all necessary active substances, such as siderophores and organic acids. If the GMG378 strain has reduced biofilm-forming ability due to *LuxR* mutations, this would explain its lower effectiveness in plant experiments. Without biofilm formation, organic acids diffuse into the bulk medium rather than creating localized high concentrations at the mineral surface.

Interspecific genetic analysis of *E. ludwigii* and *E. soli* has also revealed differences in the *PhoE* and *LysR* genes. The *LysR* gene encodes a transcription factor belonging to the LysR-type transcriptional regulator family, one of the largest families of bacterial regulators, and is involved in regulating various metabolic processes, such as virulence, quorum sensing, and biofilm formation [32]. The presence of multiple missense mutations in the *LysR* gene in *E. soli*, with 4 substitutions and 3 changing amino acid class (Supplementary Table S4), suggests potential differences in biofilm formation regulation between these two species. It is important to note that the proposed role of biofilms as a potential mechanism for differences in regulation remains a hypothesis based on genomic data analysis and literature review, and further experimental confirmation is required.

The first limitation of our study is that we have not yet verified the expression levels of genes regulated by the TF in the studied strains under different conditions. In future studies, we plan to perform qRT-PCR analysis of LuxR target genes and genes related to biofilms under phosphate-limiting conditions. The second limitation of our study is that we did not verify the ability of the strains to colonize plants. Based on our data, we can formulate a hypothesis that the mechanism of phosphate solubilization occurs through the formation of compartments from biofilms, inside which organic acids secreted by microorganisms can work more effectively without being affected by factors such as the high buffering capacity of soil. This mechanism is likely to occur in the rhizosphere due to its nature. However, as the mechanisms of plant-microbe interactions that promote plant growth are complex, it would be advisable to conduct further studies to determine whether microorganisms remain in the rhizosphere or colonize plant tissues in the future.

## 4. Materials and Methods

### 4.1. Bacterial Strains

Three strains of *Enterobacter ludwigii* (GMG278, GMG291, and GMG378), and one strain of *Enterobacter soli* (GMG1156), which were deposited in the collection of the Institute of Chemical Biology and Fundamental Medicine of the SB RAS and previously characterized, were selected for this study. The cultivation conditions, general characteristics of the strains, phosphate solubilization, whole-genome sequencing (WGS) methodology, de novo assembly, quality control, species verification, and genome annotation were described in our previous papers [13,14].

### 4.2. Design of Experiments in Pots

The aim of the experiment was to investigate whether plants that grow and develop under conditions of nutrient deficiency can somehow compensate for this lack of nutrients. The experimental design included two types of control groups. The first control group, “minimum conditions”, was watered with water only. The second control group was watered with a complete set of mineral fertilizers, providing “full nutrition”. Two experimental groups were given an incomplete set of mineral fertilizers: one lacked phosphorus and the other lacked nitrogen. Peat was chosen as a substrate for the greenhouse experiment because it best simulates the conditions of phosphate deficiency, which can be compensated for by solubilizing insoluble forms of phosphorus. The water-soluble phosphate content in peat is negligible (2–3 mg per kg of peat), while the majority of phosphorus compounds are in the form of insoluble phosphates of Ca, Al and Fe. In the top layer of the peat, iron phosphate concentrations reach 1300 mg/100 g, aluminum phosphate up to 1250 mg/100 g and calcium phosphate up to 490 mg/100 g [33].

The effect of bacterial inoculation on plant growth was investigated using the wheat cultivar ‘Novosibirsk 31’ in a greenhouse pot experiment. Pots with a diameter of 10 cm and a depth of approximately 15 cm were used. Each pot contained 0.25 kg of peat, and five seeds were planted at a depth of 2–3 cm. All the seeds were surface sterilized with 1% sodium hypochlorite for 90 s and rinsed twice with sterile distilled water before air-drying. Bacterial cultures were grown in 50-mL centrifuge tubes containing 10 mL of LB broth and shaken at 200 rpm for 48 h. The cultures were then diluted with sterile distilled water to achieve a concentration of 10^8^ cfu/mL. Seeds were coated with the bacterial solution by immersing them in the suspension for 120 min. After draining the suspension, the seeds were left in Petri dishes for three days to germinate. After three days, the germinated seeds were then planted in peat.

The control pots were irrigated with water. In the case of adding mineral fertilizer, including nitrogen and phosphorus, watering was done using a solution in mg/L containing the following: N-103, P-37, K-111, Ca-113, Mg-27, and S-35, with the addition of chelated elements such as Fe—3.87, Mn—2.6, Zn—0.53, Cu—0.53, Mo—0.13 and B—0.5. Conditions of nitrogen or phosphorus deficiency were created by excluding the corresponding components from the mineral mixture used for irrigation. Watering was done as soon as the soil became dry. The experiment was set up as a randomized trial with three biological replicates.

The plant growth lasted for 30 days. During the growing period, the average temperature was 22 °C, and the relative humidity fluctuated between 50% and 60%. Agronomic parameters such as plant height (cm), root and aboveground biomass (g), and dried root and aboveground biomass (g) were measured after 30 days. The content of chlorophyll a and b, carotenoids were determined as described in [34] (extraction method in 95% ethanol), and the proline content was determined as described in [35] (Colorimetric Assay).

Comparing the groups for statistical differences in these data, we tested the significance using ordinary two-way ANOVA analysis and Tukey’s multiple comparisons test, as implemented in GraphPad Prism 10 (https://www.graphpad.com/features, accessed on 10 September 2025). A principal component analysis (PCA) was performed to analyze the relationships between soil isolates and parameters measured by various tests. PCA was carried out using the R prcomp() function with default parameters (https://www.rdocumentation.org/packages/stats/versions/3.6.2/topics/prcomp, accessed on 10 September 2025). The results were presented on a two-dimensional plot, where the axes are the first two principal components (PC1 and PC2). The interpretation of this plot is based on the clustering of samples into different groups.

### 4.3. Phosphate Solubilization Mechanisms of the Strains

The phosphate solubilization potential of bacterial strains was investigated using three insoluble phosphate sources: tricalcium phosphate (Ca_3_(PO4)_2_), aluminum phosphate (AlPO_4_), and iron phosphate (FePO_4_) in a buffered medium. NBRIP medium with the following micronutrients was used for cultivation (mg/L): ferrous sulfate (FeSO_4_)—3.5; zinc sulfate (ZnSO_4_)—0.16; copper sulfate (CuSO_4_)—0.08; boric acid (H_3_BO_3_)—0.5; calcium chloride (CaCl_2_)—0.03; manganese sulfate (MnSO_4_)—0.4, buffered with 100 mM Tris-HCl at pH 8.0. The medium was supplemented with either 0.5% (*w*/*v*) of each of the three phosphate sources and incubated at 30 °C for 7 days under shaking conditions. An uninoculated control was also included. All experiments were conducted in triplicate. After incubation, the supernatant was separated from bacterial cells by centrifugation at 10,000 rpm for 10 min. All subsequent measurements were performed on the supernatant. The pH of the culture supernatant was determined. Phosphate solubilization was quantified using the standard method described by Murphy and Riley [36].

The determination of organic acids produced by the strains was performed using HPLC. Before applying the samples to HPLC, the supernatants were prepared by filtering through a 0.45 µm nylon membrane and precipitation of proteins and DNA, which helped eliminate background signal and non-specific peaks. Twenty microliters of the cleaned supernatant were injected into the HPLC system (Chromatec Crystal 2014 HPLC system [Chromatec, Yoshkar-Ola, Mariy El, Russia]). The organic acids were separated using a Symmetry C18 column (5.0 µm, 4.6 × 250 mm^2^, Waters Corporation, Milford, MA, USA), with a mobile phase consisting of 0.008 M H_2_SO_4_ and a mixture of 1 mM sulphuric acid, 8 mM sodium sulphate, and 0.1% orthophosphoric acid. The flow rate was set at 0.5 mL/min. The organic acids present in the culture filtrate were quantified at 210 nm using standards for different organic acids: oxalic, tartaric, formic, malic, malonic, lactic, and citric. After identifying malic acid as the main secreted acid, we discovered that HPLC detected not pure malic acid but a complex with a corresponding metal. To accurately measure the amount of malic acid, we constructed quantitative standards for malic acid complexes with iron, aluminum, and calcium. The study of malic acid secretion levels depending on cultivation conditions was conducted by culturing strains for 1 day in two media types: LB and NBRIP. After similar sample preparation, the quantities were measured using HPLC. Statistical differences in the data were compared using ordinary two-way ANOVA and Tukey’s multiple comparison tests using GraphPad Prism 10 software.

### 4.4. Comparison of Complete Genome Sequences

In order to identify the causes of the differences in the levels of secreted malic acid and detected free phosphate ions, we conducted a syntenic analysis of the genomes of the strains we studied. The genomes of these strains had previously been sequenced, annotated, and made publicly available [13,14]. These genome sequences were deposited at DDBJ/ENA/GenBank and are available under the accessions listed in Table 3. The *Enterobacter ludwigii* strain EN-119 was used as the reference strain.

**Figure 8:**
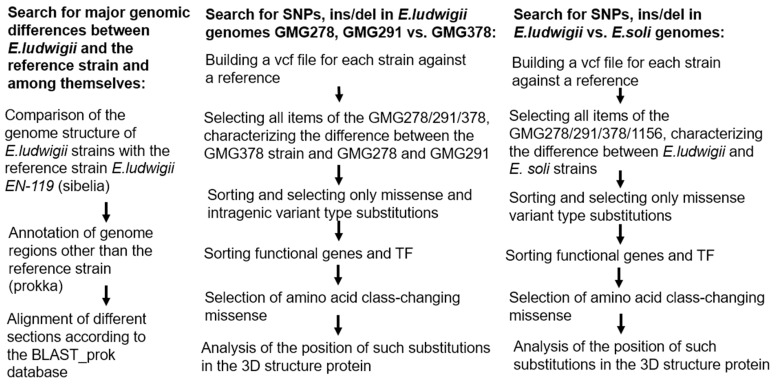
Scheme of the syntenic analysis of genomes.

Phenotypic differences between strains of *Enterobacter ludwigii* can be caused by both large genomic rearrangements, such as the presence or absence of unique genes involved in biochemical processes like phosphate solubilization, as well as by single substitutions or small insertions and deletions in key genes or their regulatory regions. In order to identify the specific causes of these differences in phosphate solubilization activity and their impact on wheat growth and development, we conducted a comparative analysis between different *Enterobacter ludwigii* strains. Additionally, a comparative analysis of the genomes of *Enterobacter ludwigii* and *Enterobacter soli*, was carried out to identify interspecific differences that may contribute to the observed differences in phosphate solubilization capabilities. The genomes were analyzed according to the scheme in Figure 8.

Comparison of complete genomes of *Enterobacter ludwigii* species strains was performed to find synteny blocks using Sibelia v3.0.7 (available at: https://github.com/bioinf/Sibelia, accessed on 15 September 2025) [37] and subsequent visualization of the results using Circos v0.52 (available at: http://circos.ca, accessed on 15 September 2025) [38]. Annotation of the target region of the genome was done using the Prokka v1.14.6 (available at: https://github.com/tseemann/prokka, accessed on 15 September 2025) [39]. Pairwise local alignment of the target region was done by BLASTn v2.12.0+ (NCBI, Bethesda, MD, USA), against the database prok_nt. The search for single-nucleotide polymorphisms (SNPs) and short insertions/deletions between complete genomes was done using Snippy v4.0.2 (available at: https://github.com/tseemann/snippy, accessed on 15 September 2025), and the identified substitutions were then compared using bcftools v1.14 [40].

## 5. Conclusions

In total, we have demonstrated that the main mechanism of phosphate solubilization in *Enterobacter ludwigii* and *soli* is the formation of metal complexes with malic acid, resulting in the release of phosphate ions. Iron phosphate appears to be a more effective source of phosphate, likely due to the involvement of a second mechanism—the formation of iron complexes with siderophores. HPLC analysis has revealed a significant reduction in the secretion of organic acids in a nutrient-rich medium. Genetic analysis of *Enterobacter ludwigii* strains has revealed differences in the structure of the *LuxR* transcription factor gene, resulting in amino acid substitutions in regions of predicted active sites. LuxR regulates biofilm formation in response to phosphorus starvation. Therefore, the phenotypic differences in phosphate solubilizing ability, confirmed by screening, HPLC, and plant experiments, are likely due to a reduced ability to form biofilms in the GMG378 strain.

The patterns we found suggest that the search for phosphate immobilization strains from inorganic phosphate should also include screening for their ability to form biofilms and HPLC analysis for the secretion of organic acids under different cultivation conditions.

## Figures and Tables

**Table 1:** The stability constant of malate complexes with Fe, Al, Ca.

	Me:Malat Ratio	logK_1_	logβ_2_	pKsp (Me-P)	Ref.
Al (III)	1:1	4.519 ± 0.019		5.75 × 10^−19^	[23,24]
Fe (III)	1:2	12.66 ± 0.33	15.21 ± 0.25	1.29 × 10^−22^	[24,25]
Ca (II)	1:1	2.25 ± 0.31		2 × 10^−29^	[24,26]

**Table 2:** Siderophore gene presence in *E. ludwigii* strains GMG278, GMG291, and GMG378, and *E. soli* strain GMG1156.

Gene Name	Gene Annotation	KEGG_DB	GMG278, GMG291, GMG378	GMG1156
*exbB*	biopolymer transport protein ExbB	K03561	+	+
*exbD*	biopolymer transport protein ExbD	K03559	+	+
*tonB*	periplasmic protein TonB	K03832	+	+
*entD*	Enterobactin synthase component D	K02362	+	+
*entF*	Enterobactin synthase component F	K02364	+	+
*entS*	enterobactin (siderophore) exporter	K08225	+	+
*entC*	isochorismate synthase	K02361	+	+
*entE*	Enterobactin synthase component E	K02363	+	+
*entB*	Enterobactin synthase component B	K01252	+	+
*entA*	2,3-dihydro-2,3-dihydroxybenzoate dehydrogenase	K00216	+	+
*fepA*	Ferrienterobactin receptor	K19611	+	+
*fepB*	Ferric enterobactin-binding periplasmic protein FepB	K23185	+	+
*fepC*	Ferric enterobactin transport ATP-binding protein FepC	K23188	+	+
*fepD*	fepD, fagA, cchC, desH; iron-siderophore transport system permease protein	K23186	+	+
*fepG*	fepG, fagB, cchD, desG; Ferric enterobactin transport system permease protein	K23187	+	+
*fes*	iron(III)-enterobactin esterase	K07214	+	+
*fecB*	Fe^3+^ dicitrate-binding periplasmic protein FecB	K23181	No	+
*fecC*	Fe^3+^ dicitrate transport system permease protein FecC	K23183	No	+
*fecD*	Fe^3+^ dicitrate transport system permease protein FecD	K23182	No	+
*fecE*	Fe^3+^ dicitrate transport ATP-binding protein FecE	K23184	No	+

**Table 3:** DDBJ/ENA/GenBank under the access number.

Internal Code of Strain	NCBI Name	Version Described in This Paper
GMG278	*Enterobacter ludwigii* strain AF137-NN-B1	JBHGBZ000000000.1
GMG291	*Enterobacter ludwigii* strain AF137-PP-C2	JBHGCA000000000.1
GMG378	*Enterobacter ludwigii* strain AF-SC-P-D6.1	JBHGCC000000000.1
GMG1156	*Enterobacter soli* strain AF-22b-4245	JBHGCI000000000.1
Reference for *E. ludwigii*	*Enterobacter ludwigii* strain EN-119	GCF_001750725.1

## Data Availability

The original contributions presented in this study are included in the article/Supplementary Materials. Further inquiries can be directed to the corresponding authors. Whole Genome Shotgun project of *Enterobacter soli* AF-22b-4245 has been deposited at DDBJ/ENA/GenBank under the accession JBHGCI000000000; *Enterobacter ludwigii* strain AF137-NN-B1 under the accession JBHGBZ000000000.1; *Enterobacter ludwigii* strain AF137-PP-C2—JBHGCA000000000.1; *Enterobacter ludwigii* strain AF-SC-P-D6.1—JBHGCC000000000.1.

## Supplementary Materials

The supporting information can be downloaded at: https://www.mdpi.com/article/10.3390/ijms27010322/s1.

## Abbreviations

The following abbreviations are used in this manuscript:

Al	aluminum
Ca	calcium
Fe	iron
PSM	Phosphate-solubilizing microorganisms
HPLC	High performance liquid chromatography

## Appendix A

**Table A1:** The growth and biochemical characteristics of wheat plants grown under conditions of lack of nutrients.

Nutrition	Bacteria	Plant Height (cm)	Leaf Fresh Weight (g)	Root Fresh Weight (g)	Leaf Dry Weight (g)	Root Dry Weight (g)	Chl A/Chl B
Water	No	18.9 ± 1.4	0.35 ± 0.02	0.65 ± 0.12	0.17 ± 0.03	0.18 ± 0.02	1.83 ± 0.12
Water	GMG278	21.2 ± 0.8	0.39 ± 0.04	0.73 ± 0.04	0.16 ± 0.01	0.23 ± 0.03	1.78 ± 0.11
Water	GMG291	21.3 ± 0.3	0.36 ± 0.07	0.79 ± 0.15	0.17 ± 0.01	0.23 ± 0.01	1.97 ± 0.06
Water	GMG378	20.2 ± 0.2	0.41 ± 0.02	0.56 ± 0.05	0.15 ± 0.04	0.18 ± 0.04	1.87 ± 0.07
Water	GMG1156	18.8 ± 1.3	0.44 ± 0.03	0.88 ± 0.05	0.19 ± 0.05	0.24 ± 0.01	1.41 ± 0.30
def N	No	23.6 ± 0.6	0.53 ± 0.09	0.72 ± 0.05	0.18 ± 0.04	0.23 ± 0.04	1.90 ± 0.03
def N	GMG278	30.0 ± 2.5	0.69 ± 0.08	0.83 ± 0.09	0.25 ± 0.08	0.24 ± 0.05	1.96 ± 0.02
def N	GMG291	25.2 ± 2.8	0.76 ± 0.08	0.91 ± 0.10	0.25 ± 0.04	0.35 ± 0.07	2.00 ± 0.07
def N	GMG378	23.6 ± 0.9	0.56 ± 0.03	0.92 ± 0.13	0.22 ± 0.03	0.31 ± 0.04	1.84 ± 0.02
def N	GMG1156	22.9 ± 1.4	0.55 ± 0.06	0.99 ± 0.13	0.20 ± 0.03	0.28 ± 0.04	1.96 ± 0.10
def P	No	32.8 ± 2.2	1.13 ± 0.08	1.36 ± 0.09	0.38 ± 0.12	0.46 ± 0.06	1.91 ± 0.02
def P	GMG278	42.3 ± 3.3 **	1.71 ± 0.01 **	1.53 ± 0.23	0.66 ± 0.04 *	0.51 ± 0.10	1.76 ± 0.09
def P	GMG291	45.2 ± 4.0 ***	1.74 ± 0.30 ***	1.57 ± 0.19	0.53 ± 0.16	0.76 ± 0.02 *	1.71 ± 0.16
def P	GMG378	38.8 ± 7.1	1.35 ± 0.07	1.33 ± 0.24	0.58 ± 0.16	0.51 ± 0.10	1.79 ± 0.05
def P	GMG1156	46.2 ± 2.8 ****	1.59 ± 0.23 *	1.65 ± 0.26	0.59 ± 0.09	0.64 ± 0.06	1.75 ± 0.06
NP	No	38.9 ± 2.9	2.05 ± 0.24	1.82 ± 0.21	0.69 ± 0.01	0.68 ± 0.08	1.29 ± 0.20
NP	GMG278	53.7 ± 0.8 ****	2.59 ± 0.42 **	2.06 ± 0.16	1.21 ± 0.23 ****	0.76 ± 0.15	1.03 ± 0.29
NP	GMG291	45.9 ± 4.0	2.84 ± 0.28 ****	2.11 ± 0.17	1.11 ± 0.24 ***	0.71 ± 0.04 *	0.91 ± 0.28
NP	GMG378	48.8 ± 2.8 **	2.13 ± 0.28	1.79 ± 0.16	0.78 ± 0.17	0.75 ± 0.14	1.14 ± 0.32
NP	GMG1156	48.1 ± 8.2 *	2.47 ± 0.10 *	1.85 ± 0.35	1.04 ± 0.18 **	0.67 ± 0.06	1.07 ± 0.31

The statistical significance of the differences in these characteristics was calculated within each group (Water, def N, def P, NP) relative to the control within this group (without inoculation). * *p* < 0.05, ** *p* < 0.01, *** *p* < 0.001, **** *p* < 0.0001.

**Table A2:** Evaluation of phosphate solubilization from insoluble forms of phosphates in vitro.

Strain	AlPO_4_			Ca_3_(PO_4_)_2_			FePO_4_		
	pH	Soluble P, µg/mL	MA, µg/mL	pH	Soluble P, µg/mL	MA, µg/mL	pH	Soluble P, µg/mL	MA, µg/mL
*E. ludwigii* GMG278	4.07 ± 0.09	60.8 ± 1.7	9415.8 ± 778.9	3.68	23.0 ± 7.34	4559.4 ± 451.0	2.76	95.9 ± 3.9	2761.2 ± 194.4
*E. ludwigii* GMG291	3.38 ± 0.12	78.8 ± 4.6	7042.4 ± 742.3	3.72	48.7 ± 15.6	6898.4 ± 239.8	2.65	95.9 ± 0.4	2407.7 ± 15.0
*E. ludwigii* GMG378	3.41 ± 0.18	81.3 ± 4.4	3980.3 ± 533.3	4.04	56.4 ± 11.6	5142.2 ± 587.5	2.71	97.7 ± 5.3	1602.3 ± 47.0
*E. soli* GMG1156	3.47 ± 0.14	82.8 ± 3.0	29,119.2 ± 197.1	4.23	61.5 ± 14.7	22,715.0 ± 327.6	2.66	97.3 ± 5.1	10,103.1 ± 305.1

MA—malic acid.

**Table A3:** The amount of malic acid in bacterial suspension secreted by *Enterobacter* depending on the culture medium. MA—malic acid.

Strain	MA in Minimal Medium, µg/mL	MA in LB Medium, µg/mL
*E. ludwigii* GMG278	10,293.5	4606.1
*E. ludwigii* GMG291	21,591.8	3924.4
*E. ludwigii* GMG378	19,132.2	3066.6
*E. soli* GMG1156	14,816.4	5645.4
